# Anti-leucine-rich glioma-inactivated 1 encephalitis revealed by a manic episode: insights from frontal lobe dysfunction in neuropsychiatry through neuropsychology and metabolic imaging. A case report

**DOI:** 10.3389/fpsyt.2023.1168302

**Published:** 2023-05-18

**Authors:** Federica Porpiglia, Maxime Guillaume, Evangeline Bliaux, Dimitri Psimaras, Pierre Decazes, Olivier Guillin, Maud Rothärmel, Alexandre Morin

**Affiliations:** ^1^Department of Psychiatry, Rouvray Hospital, University of Rouen, Rouen, France; ^2^Department of Neurology and CNR-MAJ, CHU Rouen, Univ Rouen Normandie, UNIROUEN, Rouen, France; ^3^Department of Neurology 2-Mazarin AP-HP, Groupe Hospitalier Pitié-Salpêtrière, Paris, France; ^4^Department of Nuclear Medicine, Centre Henri-Becquerel, Rouen, France

**Keywords:** anti-LGI1 encephalitis, limbic encephalitis, autoimmune manic syndrome, FDG-PET, behavior

## Abstract

**Background:**

Anti-leucine-rich glioma-inactivated 1 (LGI1) encephalitis is a limbic encephalitis that rarely presents as an isolated psychiatric syndrome.

**Case presentation:**

A 70-year-old patient first presented with behavioral disorder including hyperactivity, euphoria, with disinhibition and accelerated speech associated with severe insomnia and cognitive disorder. A manic episode was diagnosed and he received various psychotropic medications with no improvement. Invesitgations were negative (MRI showed T2 aspecific hyperintensities with no hyperintensities in limbic regions and EEG was normal). He was transferred to a nursing home, with a diagnosis of neurodegenerative condition. Later, he was referred to our unit for further investigations. A cerebral 18F-FDG-PET revealed an association of frontal hypometabolism and temporal and striatum hypermetabolism and CSF analysis revealed slightly increased white blood cell counts. Plasmatic anti-LGI1 antibodies were detected. The patient was treated with intra-venous immunoglobulin (IvIg) but showed no improvement. Second-line treatment (a combination of rituximab and cyclophosmphamide) was then administered for a year, leading to an improvement of neuropsychiatric symptoms and normalization of metabolic impairment on 18F-FDG-PET.

**Conclusion:**

In this report, we describe a novel case of a patient withanti-LGI1 encephalitis with a predominant long-term psychiatric presentation. An atypical presentation (such as atypical psychiatric symptoms, neurocognitive disorder, and hyponatremia) should prompt further investigations such as CSF analysis, considering that MRI and EEG may be normal. FDG-PET might be of interest but few data are available in the literature. Early treatment of anti-LGI1 encephalitis is crucial for overall prognosis and may delay the development of dementia in some cases.

## Background

Limbic encephalitis (LE; also known as antibody-mediated encephalitis) represents a group of conditions which present essentially with neuropsychiatric symptoms (1). Initial presentation can vary and is often aspecific. In particular, psychiatric-onset LE can blur a clinician's judgement leading to a misdiagnosis, delayed immunotherapy and poor prognogis (2).

Although psychiatric symptoms are observed in 90% of patients (3), they often mask neurological manifestations, making the diagnosis of this disease challenging (2, 4–6). Patients are often first diagnosed with psychiatric disease, thus delaying immunotherapy.

Therefore, the aim of this report was to describe a case of anti-leucine-rich glioma-inactivated 1 (anti-GLI1) encephalitis with a predominant psychiatric presentation that caused a delay in diagnosis and treatment and a chronification of cognitive impairment.

## Case report

A 70-year-old man presented to our unit for evaluation of behavioral and cognitive disturbance.

The patient was married and he had two children. Previously, he had worked in a heating company, but he was retired at the time of the visit. Prior to disease onset, he was totally self-sufficient.

The patient's medical history showed no evidence of psychiatric or neurological conditions beforehand. At the age of 57, he had colorectal cancer, which was in remission (annual colonoscopy). At the age of 67 (August 2018), he developed various behavioral symptoms such as hyperactivity, euphoria, with disinhibition and accelerated speech, and he had many renovation projects for his house. Moreover, he suffered from severe insomnia and reduced cognitive performance (for example, he was not able to recognize his own home or to remember dates). In October 2018, he started to present hallucinations, such as a sensation of bugs moving on his legs, seeing people in the woods, and hearing the phone ringing constantly. He also presented with persecutory delusion. At the same time, several episodes of confusion and temporospatial disorientation were reported by his wife. By December 2018, he started to exhibit cognitive impairment and memory loss together with insomnia and psychomotor agitation.

In January 2019, he was admitted to the psychiatric department of Dieppe Hospital (Normandy). Behavioral symptoms such as psychomotor agitation, insomnia and persecutory delusions were predominant. A diagnosis of manic episode with psychotic symptoms was discussed and further investigations were performed due to the atypical presentation. Magnetic resonance imaging (MRI) showed aspecific white matter hyper intensities, electroencephalogram (EEG) was normal and no neurological diagnosis was retained. At this time, laboratory testing showed hyponatremia (i.e., 132 mmol/L; reference range 136–145 mmol/L). The patient was treated with risperidone 2 mg but he experienced severe side effects such as confusion and hypothermia. He was then treated with sodium valproate 1 g.

Despite neuroleptic and mood-regulating treatments, the patient showed no improvement in clinical presentation whilst cognitive symptoms worsened. In August 2019, he was discharged with a diagnosis of dementia and was transferred to a nursing home.

Because of the severity of the disease a new visit was scheduled. In December 2019, the patient was admitted to our memory clinic at Rouen University Hospital, 1½ year after symptom exacerbation.

The neuropsychiatric examination pointed to mood fluctuations, apathy, delusions with a prominent Capgras syndrome, and insomnia. The patient did not present hallucinations or suicidal tendencies and had normal appetite. On the other hand, severe attention disorder, disinhibition, episodic memory impairment together with severe temporospatial disorientation and illogical speech were detected.

Neurological examination revealed no pyramidal or extrapyramidal syndrome, no sensorimotor disorder, no dysarthria, no facio-brachial dystonic seizures, no sign of dysautonomia, no ataxia.

Cerebrospinal fluid (CSF) analysis showed mildly elevated leukocytes (9/uL, normal range 0–8/uL) and an elevated protein level (49 mg/dL, normal range 20–40 mg/dL). Anti-LGI1 antibodies were positive both in serum and CSF. Sodium level and other laboratory blood tests were within normal ranges. Multiple EEGs including one sleep-deprived EEG were performed with no epileptiform discharges or patterns suggesting encephalitis. Cranial MRI exhibited few aspecific hyperintensities on T2-weighted fluid-attenuated inversion recovery sequences with no hyperintensities in limbic regions.

**Figure 1:**
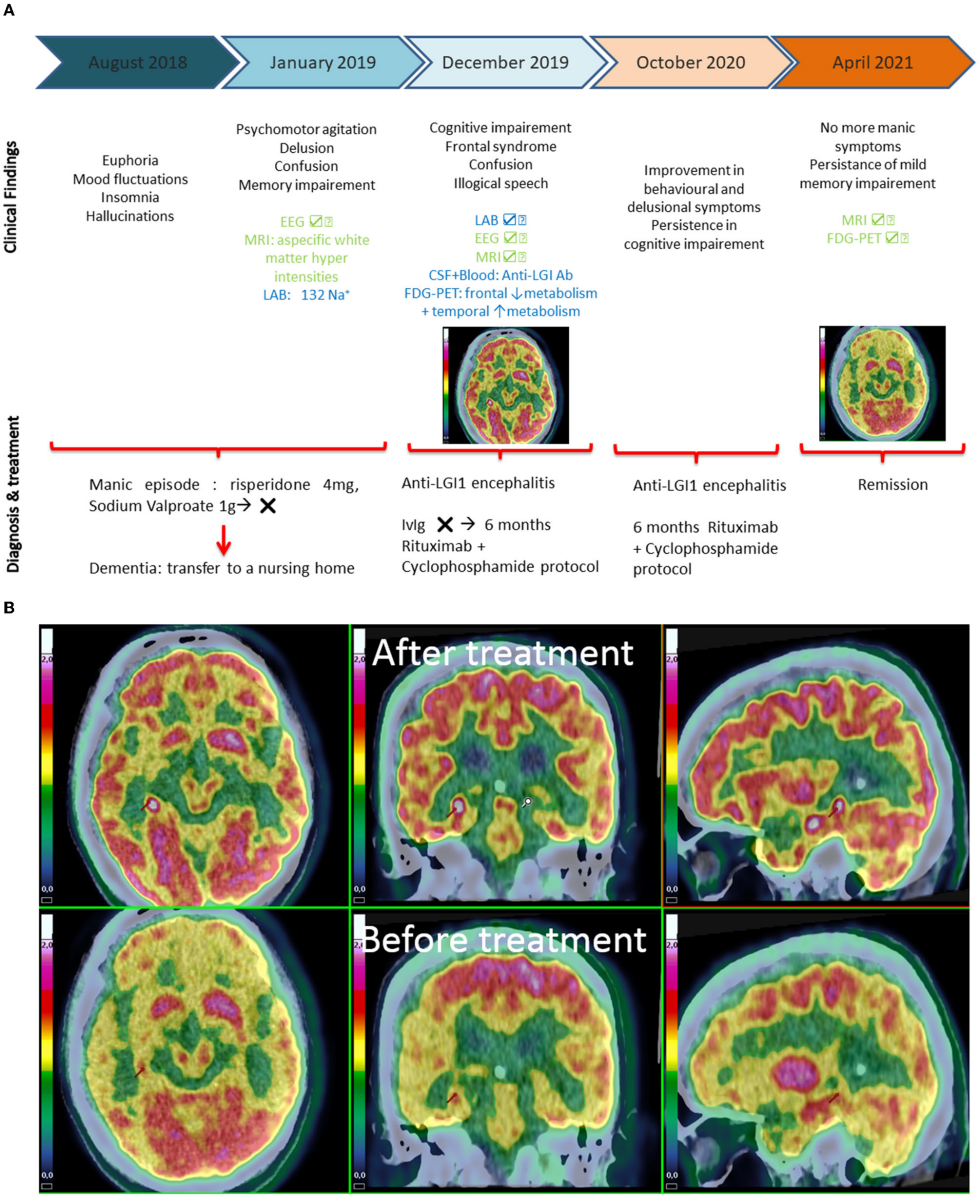
**(A)** Timeline from first symptoms to diagnosis and final treatment. **(B)** Brain FDG-PET: axial, coronal and sagittal orientation. Before treatment: diffuse frontal and temporal hypometabolism and striatum hypermetabolism. After treatment: improvement of orbito-frontal lobe and temporal hypometabolism and, to a smaller extent, of dorsolateral lobe hypometabolism. Anatomical orientation (right hemisphere on the right part of the figure). SUV ranging from 0 (blue) to 2 (red).

A cerebral FDG-PET examination revealed the association of frontal hypometabolism and temporal and striatum hypermetabolism (Figure 1). A whole-body FDG-PET/computer tomography scan revealed no structural or metabolic abnormalities.

Neuropsychological testing showed a deficit in Mini Mental State Examination (24 out of a maximum of 30 points), frontal syndrome [Frontal Assessment Battery (7) = 12/18] and severe difficulties in episodic memory (8, 9) as presented in Table 1.

**Table 1:** Neuropsychological assessment before treatment (March 2019 and January 2020) and after treatment (October 2020).

	**Neuropsychological test**	**Maximum**	**Mar-19**	**Jan-20**	**Oct-20**	**N/P**
Global cognition	MMSE	30	24	18	20	**P**
Praxis	Gestures	8	8	7	8	N
Visuo-constructive skills	Rey's figure (copy)	36	36	35	33	N
Episodic memory	Free and cued selected recall test	list	A	A	B	
	Immediate recall	16	8	Aborted	14	N
	Free recall 1	16	2		4	**P**
	Total recall 1	16	2		8	**P**
	Free recall 2	16	0		4	**P**
	Total recall 2	16	Aborted		11	**P**
	Free recall 3	16			5	**P**
	Total recall 3	16			8	**P**
	Delayed free recall	16			2	**P**
	Delayed total recall	16			3	**P**
Frontal lobe dysfunction	Frontal Assesment Battery	18	12	13	12	N
	Trail Making Test					
	Trail Making Test A		46		64	N
	Trail Making Test B		223		95	N
	Trail Making Test Errors A		0		0	N
	Trail Making Test Errors B		0		0	N
Language	Naming	80	79	78	78	N
	Verbal fluency					
	Phonologic fluency		14	7	20	N
	Semantic fluency		11	5	16	N

N, normal range; P, pathological range. Bold values means “pathological scores”.

The patient was diagnosed with anti-LGI1 autoimmune encephalitis and was initially treated with intra-venous immunoglobulins (IvIg) but showed no improvement of neuropsychiatric symptoms. A second-line treatment was then proposed to the patient, and he was administered immunotherapy combining rituximab and cyclophosphamide (rituximab on Day 1 (D1) and D15, cyclophosphamide on D30 and then once a month).

In October 2020, 6 months after the beginning of treatment, neuropsychiatric examination showed an improvement of behavioral and delusional symptoms but only a mild improvement of cognitive impairment: progress in memory tests but persistency of frontal lobe syndrome (Table 1).

Immunotherapy was pursued for 6 months followed by a rituximab perfusion twice a year with no adverse event.

New assessments were performed in April 2021, 1 year after the beginning of treatment. Clinical evaluation showed a consistent improvement of psychiatric symptoms, with normal thought and attention, no more mood fluctuations, disinhibition or apathy, no hallucinations or delusion. Sleep disturbances were still present, and a specialized examination ruled out obstructive sleep apnea (OSA). Cerebral FDG-PET showed a normalization of metabolic impairment (Figure 1).

## Discussion

This report outlines the case of a patient presenting chronified anti-LGI1 encephalitis who had exhibited predominant manic syndrome with atypical symptoms including cognitive impairment for 1½ years. Our patient showed an obvious recovery after 1 year of immunomodulating treatment, from both a clinical and a metabolic point of view.

The predominant psychiatric manifestation of anti-LGI1 encephalitis over the course of almost 2 years without brachio-facial seizures or other neurological symptoms apart from cognitive disorder is rare. The clinical features of the present case are essentially those of a manic episode with insomnia, disinhibition, psychomotor agitation, flight of ideas and delusion. Neurocognitive symptoms, which are present in 97% of cases (10), appeared secondarily.

Classically, insomnia is more associated with a psychiatric condition, but it is also common in LGI1 encephalitis. This could have been misleading and could explain why no lumbar puncture or FDG-PET was proposed for the patient.

Disinhibition, agitation, working memory deficits can point to a frontal lobe syndrome as encountered in neurological conditions, but is also common in manic episodes (11). Cognitive disorders can sometimes be misleading in psychiatric conditions. It is often difficult to disentangle neurological from psychiatric frontal lobe dysfunction during the acute manic phase. Nevertheless, cognitive disorder should not evolve negatively in bipolar disorder, apart from long-term bipolar disorder (12) which was not the case here. Furthermore, episodic memory was involved which is more specific to neurological conditions.

Thus, rapidly evolving cognitive disorders, even when associated with psychiatric symptoms, should prompt extensive neurological workup. Most frequent diagnoses are sporadic Creutzfeldt-Jakob disease, autoimmune encephalitis and require MRI, EEG and lumbar puncture (13).

Three cases of patients presenting anti-LGI1 encephalitis with psychiatric presentation have been published, two with psychotic symptoms and memory loss (one of whom presenting faciobrachial seizures) (4, 14), and one with manic syndrome. However, unlike two of the above-cited cases (14), our patient's atypical presentation led to a delayed diagnosis as he presented symptoms for almost 2 years before being treated. Secondly, he did not have neurological symptoms and, in particular, he did not experience epileptic seizure, a feature which motivated clinicians to perform further tests in the above-cited case (15). Finally, our patient did not respond to first-line treatments but improved considerably after 1 year of rituximab-endoxan protocol, as opposed to one of these cases.

The concept of “autoimmune psychosis,” including schizophrenia and manic episodes, was recently suggested, and red flags and consensus statements have been published (6). In this case, there were three red flags for a hidden autoimmune encephalitis: firstly, the atypical psychiatric presentation associated with severe cognitive impairment; secondly, the atypical age of onset of psychiatric symptoms, bipolar disorder being mostly diagnosed at a young age; and finally the clinical deterioration despite psychopharmacological treatment.

The positivity of anti-LGI1 antibodies in serum and CSF together with metabolic impairment revealed on FDG-PET led to a final diagnosis of anti-LGI1 encephalitis. The EEG of the present case is rare in this context (16). However, typical electro encephalographic features have not been described in the literature (17), and our patient did not have epileptic seizures which could explain the absence of EEG impairment. Hyponatremia, an emblematic feature of anti-LGI1 encephalitis, was initially observed. It has been reported in 60% of patients suffering from the disease, although the underlying mechanism has been investigated in a limited number of patients (18).

Meaningfully, cerebral FDG-PET findings were consistent with the association of temporal or basal ganglia hypermetabolism and frontomesial hypometabolism as described in other auto-immune encephalitis (18–20). Two case reports identified the presence of hypermetabolism in the basal ganglia as well as in the left hippocampus and amygdala in their patients a few months after clinical onset of anti-LG1 encephalitis (8, 21). In addition, Shin et al. showed temporal and bilateral basal ganglia hypermetabolism in most of their patients (3 days to 2 years further to diagnosis), suggesting that the anti-LGI1-induced brain metabolic pattern may depend on the disease evolution and delay between clinical onset and treatment administration.

Moreover, our patient's clinical improvement assessed 1 year after the beginning of the treatment was congruent with the disappearance of metabolic impairment on FDG-PET.

Our patient's poor response to anti-inflammatory first-line medications may be associated with the delayed diagnosis and therefore the start of treatment, as it is well-known that early medication is associated with better prognosis in autoimmune encephalitis (22). This concept is even more outstanding when considering anti-LGI1 encephalitis, as it is rarely associated with cancer comorbidities and has for this reason a better long-term prognosis if treated rapidly (10).

A recently published case report introduced the concept of a difference between “acute inflammatory state” and “state of organ damage” regarding autoimmune encephalitis (4). In the first case, anti-inflammatory treatment can control or completely suppress disease activity and thus possibly prevent irreversible damage, as opposed to the “state of organ damage” in which organ dysfunction has already occurred due to inflammatory activity. In the present case, the patient might have suffered from both lesions.

Some inflammatory lesions with altered metabolism on FDG PET might have been medication-responsive, such as behavioral symptoms (related to the orbito-frontal region) and improved memory impairment (related to the mesial temporal region). Some damage might have been out of reach of immunotherapy, especially regarding executive dysfunction (related to the dorso-lateral cortex), which remained impaired after 1 year of treatment.

Moreover, the severity of disease presentation required not only to use a second-line protocol, but also to extend the treatment period from 6 months to a year. Such treatments are not without side effects and the state of induced immunodeficiency could potentially lead to other infectious diseases. Future studies should focus on identifying biomarkers or clinical markers (red flags) of “acute inflammatory state” in predominantly psychiatric forms of anti-LGI1 encephalitis, in order to promote early treatment and therefore prevent the “state of organ damage.”

## Conclusion

A predominantl neurocognitive and manic presentation of anti-LGI1 encephalitis can lead to delayed diagnosis and treatment (2), therefore inducing a poorer response to medication. The detection of red flags for the presence of autoimmune encephalitis in patients with atypical psychiatric presentation is essential to treat the disease as rapidly as possible and to achieve full recovery. In this report, we describe a novel case of a patient with chronified anti-LGI1 encephalitis which improved both clinically and radiologically after 1 year of second-line treatment.

## Patient's perspective

The patient was relatively anosognosic and did not express any improvement or side effects. His spouse reported a major subjective improvement of behavior and cognition and was prone to pursue intensive therapeutics.

## Data availability statement

The original contributions presented in the study are included in the article/supplementary material, further inquiries can be directed to the corresponding author.

## Ethics statement

Written informed consent was obtained from the individual(s) for the publication of any potentially identifiable images or data included in this article.

## Author contributions

AM and FP: substantial contributions to the conception or design of the work and drafting the work. AM, FP, EB, DP, and PD: acquisition, analysis, and the interpretation of data for the work. FP, MG, DP, PD, OG, and MR: revising the work critically for important intellectual content. AM: final approval of the version to be published. All authors contributed to the article and approved the submitted version.
